# Influencing factors of cancer prevention and control among urban and rural adults in Fujian, China: A cross-sectional survey

**DOI:** 10.3389/fpubh.2022.1053183

**Published:** 2022-12-21

**Authors:** Tian Bao Yang, Xiu Jing Lin, Jia Ling Lin, Wei-Ti Chen, Fei Fei Huang

**Affiliations:** ^1^Department of Cardiothoracic Surgery, Affiliated Hospital of Putian University, Putian, China; ^2^School of Nursing, Fujian Medical University, Fuzhou, China; ^3^School of Nursing, University of California, Los Angeles, Los Angeles, CA, United States

**Keywords:** awareness, cancer prevention, core knowledge, survey, China

## Abstract

**Objective:**

Cancer burden can be reduced when the population's knowledge of cancer prevention and control measures is increased. However, current epidemiological research investigating cancer prevention and control knowledge in China is limited. This study aimed to examine the core knowledge levels of cancer prevention and control measures as well as its influencing factors among adults in Fujian, China.

**Study design:**

A cross-sectional study.

**Methods:**

From September to December 2021, a total of 2,440 Chinese urban and rural adults from Fujian Province, located in Southeastern China, were randomly selected for this cross-sectional study. The probability proportionate approach to sampling was used. A 38-item questionnaire that covered demographics and basic knowledge of cancer, including concepts, screening, therapy, and rehabilitation-related key points was used to measure knowledge levels of cancer prevention and control measures among 2,074 participants. The level of each participants' core knowledge of cancer prevention and control measures was defined as a rate calculated by the number of correct answers divided by the total number of questions. The binary logistic regression model was used to determine if influencing factors were associated with core knowledge awareness.

**Results:**

In total, 1,290 participants (62.2%) were in the low knowledge group and 784 (37.8%) were in the high knowledge group. The average knowledge rate of cancer prevention and control measures among all participants was 56.01%. Participants from urban areas, who held white-collar jobs, were married, had a bachelor's degree or above, had a family history of cancer, or self-rated their health level as good or average were associated with higher rates of cancer prevention and control core knowledge (overall *p* < 0.05).

**Conclusion:**

These findings may assist healthcare providers and/or researchers in designing effective primary preventive interventions to enhance the general population's cancer prevention and control knowledge, and subsequently decrease the cancer burden in China.

## Introduction

Cancer is a major public health problem worldwide (1). In China, the rates of age-adjusted incidence and mortality of cancer have increased gradually since 2000 (2, 3), because of the increasing trend of urbanization and the accumulated effects of risk-factor exposure (4). However in developed countries, such as the United States and some European countries, cancer mortality rates and age-adjusted rates of cancer incidence in men have generally decreased since the early 1990s (2). Decreasing trends in cancer burden may be linked to progress in cancer research, prevention, and care in Western countries.

By some estimates, up to half of all cancer cases can be prevented or avoided (5). Since 2015, China has made efforts to confront its rapidly increasing cancer burden by implementing a series of plans and policies focused on cancer control (6, 7), in which primary and secondary prevention is always the first line of action (8). This includes improving the general population's knowledge regarding cancer prevention (5, 9). Although knowledge alone will not prevent cancer, it is necessary before one can take action (10).

Cancer prevention and control core knowledge refers to essential knowledge of actions to minimize individual cancer risk and methods used to reduce cancer burden, such as basic knowledge regarding cancer prevention, treatment, and management of the disease (5). Currently, the awareness of basic cancer prevention is reported as suboptimal, and even as low as 20% in certain Chinese populations (10–13). Li et al. (5) and Yu and Baade (14) have reported that individuals with lower levels of core prevention knowledge of cancer had more diagnoses of cancer after a median follow-up of 3.3 years. Low levels of cancer prevention core knowledge negatively impact individuals' attitudes, healthy lifestyles, and positive health behaviors (9, 15). These negative effects may be more prevalent in Eastern cultures, where people tend to reason holistically, believe in the relatedness of objects and events, and consider things to be constantly changing cyclically in everyday life (i.e., naïve dialecticism) (16).

Previous studies in China on core knowledge of cancer prevention and control measures were either conducted in a single area (urban or rural), with specific cancer populations, or with small samples, all of which limited the generalizability of their findings (5, 10–13). Reliable information on rates of core knowledge of cancer prevention and control measures, as well as its influencing factors, are required to provide further insight into what measures may be taken to reduce the heavy burden of cancer in China. This study aimed to examine the rates of core knowledge of cancer prevention and control measures as well as its influencing factors among urban and rural adults in Fujian Province, China.

## 2. Methods

### 2.1 Study design and participants

**Figure 1:**
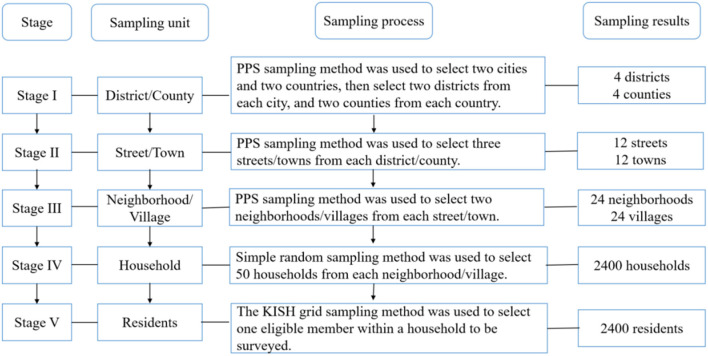
The five-stage probability proportionate to size sampling process.

From September to December of 2021, a large-scale cross-sectional study was conducted among urban and rural adults in Fujian Province, which lies on the southeastern coast of China. As of 2021, Fujian Province had a registered population of ~41.87 million within its nine major cities and rural areas. A five-stage probability proportionate to size (PPS) sampling approach was applied to select 2,440 participants. The study's recruitment process is detailed in Figure 1. Household members were eligible to participate in this study if they: (1) were locally registered residents; (2) lived in the targeted district/county for at least 6 months before the survey; (3) were aged 18–69 years old; (4) had no cognitive disorders; and (5) no cancer history.

After obtaining written informed consent, each participant was asked to complete a face-to-face interview and questionnaire regarding their core knowledge of cancer prevention and control measures and socio-demographic characteristics. This study was approved by the Ethics Review Committee of the Fujian Provincial Centers for Disease Control and Prevention (Grant number: K2021-101-01). All personally identifiable information was removed before data analysis. The study adhered to the STROBE (Strengthening the Reporting of Observational Studies in Epidemiology) statement (17).

### 2.2 Measures

The questionnaire regarding core knowledge of cancer prevention and control measures was developed by an expert panel led by the National Cancer Center of China (18, 19). The questionnaire comprised 13 single-item questions, 12 multiple-item questions, and 13 true-or-false questions that covered basic knowledge of cancer, including concepts, screening, therapy, and rehabilitation-related key points. Expert panel members assigned a numeric score to each answer provided in the study questionnaire. For the true-or-false and single-answer questions, “1 point” was assigned to a correct answer and “0 points” to an incorrect answer. For the multiple-item questions, “2 points” were assigned if the answer was exactly correct, otherwise “0 points” were assigned. The total score ranged from 0 to 50 for each participant. The total score was converted into a percentage, yielding cancer prevention and control core knowledge scores ranging from 0 to 100 points. In this study, Cronbach's α of the questionnaire was found to be 0.899.

The demographic information collected in the questionnaire included age, gender, residential area, marital status, educational level, occupation, number of family members living in the household, yearly income of the household, family history of cancer, body mass index (BMI, weight/height^2^, Kg/m^2^), smoking status, and self-evaluated health status.

### 2.3 Data analyses

Data analyses were conducted using SPSS 26.0 (IBM, Armonk, NY, USA). Approximately 5% of missing data were replaced using mean value substitution, and *p* ≤ 0.05 was considered statistically significant. The data met the assumptions of normality, with a one-sample Kolmogorov-Smirnov test yielding non-significance. Continuous variables were expressed as means with standard deviations (SDs) and categorical variables were expressed as proportions or percentages.

The level of each participants' core knowledge of cancer prevention and control measures was defined as a rate calculated by the number of correct answers divided by the total number of questions. The average knowledge rate (%) of all participants was defined as the sum of all knowledge rates divided by the total number of participants. [knowledge rate (%) = number of subjects with all correct answers/total number of subjects ^*^100.] Core knowledge levels with rates of <60% were considered to be in the low knowledge group (LKG), while rates of ≥60% were considered to be in the high knowledge group (HKG). A binary logistic regression model with a forward conditional method was used to determine the influencing factors associated with the core knowledge of cancer prevention and control measures. The outcome variable was the knowledge group and demographic variables served as independent variables. The result estimates are expressed as odds ratios (OR) (95% confidence intervals, CIs).

## 3. Results

A total of 2,440 eligible participants were recruited and 2,074 completed the survey, with a response rate of 86.42% over the 4-month period of the study. The main reasons for study drop-outs were participants had no interest or time to do the survey. No significant differences were found in age, gender, or residential area between the two knowledge level groups, and the age of participants ranged from 18 to 69 years, matching the local general population. The mean age of participants was 47.81 years (SD = 13.20), the average BMI was 23.21 kg/m^2^ (SD = 3.09), and the average number of family members was 3.56 (SD = 1.75). Table 1 shows the demographic characteristics of all participants by knowledge level group.

**Table 1:** Rates of core knowledge level by demographic characteristics of participants (*n* = 2,074).

**Variables**	**Total *n* (%)**	**HKG** **(*n* = 784) *n* (%)**	**LKG** **(*n* = 1,290) *n* (%)**
**Gender**			
Male	935 (45.1)	356 (44.1)	589 (45.7)
Female	1,139 (54.9)	428 (55.9)	701 (54.3)
**Residence area**			
Urban	1,412 (68.1)	562 (71.7)	850 (65.9)
Rural	662 (31.9)	222 (28.3)	440 (34.1)
**Marital status**			
Living alone (e.g., unmarried, divorced, and widowed)	208 (10.0)	68 (8.6)	140 (10.8)
Married	1,866 (90.0)	716 (91.4)	1,150 (89.1)
**Educational level**			
Primary school degree or below	693 (33.4)	178 (22.7)	515 (39.9)
Junior high school degree	615 (29.7)	211 (26.9)	404 (31.3)
Senior high school degree (including technical training)	415 (20.0)	194 (24.7)	221 (17.1)
Junior college diploma	205 (9.9)	124 (15.8)	81 (6.3)
Bachelor's degree or higher	146 (7.0)	77 (9.8)	69 (5.3)
**Occupation**			
White collar	296 (14.3)	171 (21.8)	125 (9.7)
Blue collar	1,438 (69.4)	441 (56.3)	997 (77.3)
Students	37 (1.8)	22 (2.8)	15 (1.2)
Unemployment	212 (10.2)	94 (12.0)	118 (9.2)
Retired	89 (4.3)	56 (7.0)	34 (2.6)
**The yearly income per household (yuan, RMB)**			
< 1,000	560 (28.2)	181 (24.6)	379 (30.3)
1,000–2,000	593 (29.9)	225 (30.6)	368 (29.4)
2,000–3,000	432 (21.8)	170 (23.1)	262 (21.0)
3,000–4,000	121 (6.1)	50 (6.8)	71 (5.7)
4,000–5,000	149 (7.5)	59 (8.0)	90 (7.2)
5,000+	130 (6.5)	50 (6.8)	80 (6.4)
**Family history of cancer**			
Yes	275 (13.3)	113 (14.4)	162 (12.6)
No	1,640 (79.1)	644 (82.1)	996 (77.2)
Don't know	159 (7.7)	27 (3.4)	132 (10.2)
**BMI (kg/m** ^2^ **)**			
< 18.5 (underweight)	97 (4.8)	41 (5.3)	56 (4.4)
18.5–24.9 (normal weight)	1,188 (58.3)	462 (60.2)	726 (57.1)
≥25 (overweight)	754 (37.0)	264 (34.4)	490 (38.5)
**Smoking status**			
Current smoker	454 (21.9)	167 (21.3)	287 (22.2)
Former smoker	133 (6.4)	42 (5.4)	91 (7.1)
Never smoked	1,487 (71.7)	575 (73.3)	912 (70.3)
**Self-evaluated health**			
Very good	876 (42.2)	310 (39.5)	566 (43.9)
Good	787 (37.9)	304 (38.8)	483 (37.4)
Average	379 (18.3)	160 (20.4)	219 (17.0)
Bad	24 (1.2)	6 (0.8)	18 (1.4)
Very bad	8 (0.4)	4 (0.5)	4 (0.3)

BMI, body mass index; LKG, low knowledge group; HKG, high knowledge group.

White-collar = office workers, teachers, healthcare providers, academic researchers, and government officials.

Blue-collar = farmer, factory worker, forestry worker, fisher, service staff, salesperson, house-worker, and driver.

### 3.1 Participants' knowledge of cancer prevention and control measures

In total, there were 1,290 participants (62.2%) in the LKG and 784 participants (37.8%) in the HKG. The average knowledge rate of cancer prevention and control measures among all participants was 56.0%. Up to half of the participants (ranging from 36.4 to 50.9%) knew the basic concepts about cancer, physical rehabilitation methods, cancer warning signs, cancer early detection methods, and cancer risk factors. More than 70.0% were aware of the meaning of early cancer detection and early treatment as well as common therapy methods (see Table 2). Regarding cancer prevention and control core knowledge items (see Supplementary Appendix A), the five lowest knowledge rates (below 30.0%) included responses to the questions: “What are breast cancer warning symptoms?” (16.5%); “Which of the following biological factors increase the risk of cancer” (22.3%); “What's the correct description of cancer prevention and therapy?” (22.7%); “Which of the following unhealthy lifestyle habits can increase the risk of cancer?” (26.7%); and “What's the correct description of cancer pain?” (27.2%). More than 70.0% of participants demonstrated basic cancer knowledge, its common treatment methods, and the meaning of early cancer detection and early treatment.

**Table 2:** Rates of cancer prevention and control core knowledge by survey domain (*n* = 2,074).

**Core knowledge domain**	**Corresponding item (s)**	**Knowledge rate (%)**
1. Cancer basic concepts	A1, C9, B1, B10, A12	47.72
2. Cancer basic knowledge	A2, B2, B5, C1	62.23
3. Cancer risk factors	A7, C5, B9, C2, A13	50.86
4. Cancer prevention measures	A5, B8, C6, C8	58.38
5. Early detection and early treatment meaning	A8, B17	73.75
6. Recognition of cancer warning signs	A9, B16, C11–12	50.15
7. Cancer early detection	B11, C10, C13	49.77
8. Timely medical treatment	A10, B19	61.50
9. Standardized treatment	A6, B20, A11	63.20
10. Following doctors' requirements to check regularly	B12, B21	68.35
11. Common treatment methods for cancer	A3	80.70
12. Physical rehabilitation	C14, C15	36.40
13. Psychological rehabilitation	A4	52.70

### 3.2 Factors associated with cancer prevention and control core knowledge

**Figure 2:**
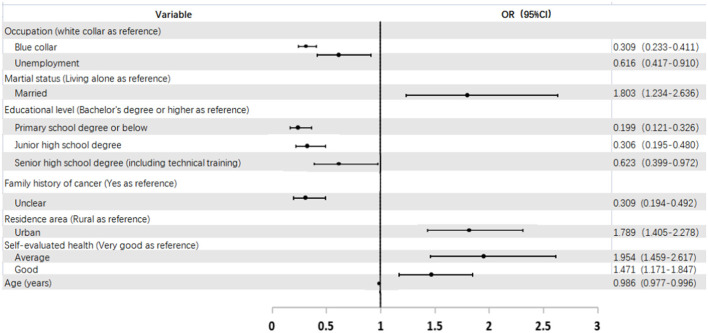
Forest plot of OR (95% CI) for having a high cancer prevention and control core knowlegde by sociodemographic and other factors in Fujian Province in China.

As shown in Table 3 and Figure 2, after adjusting for confounding factors, including gender, number of family members in the household, the yearly income of the household, BMI, and smoking status, the logistic regression analysis indicated the main predictors influencing the level of cancer prevention and control core knowledge were: residing in urban areas, being married, unclear family history of cancer, and self-evaluated average or good health. For example, participants who had an unclear family history of cancer were 0.309 times more likely to have core cancer knowledge than individuals with a known family history of cancer (OR = 0.309, 95% CI 0.194–0.492). Participants who had a blue-collar occupation, were unemployed, had a junior high school degree or below, or were older had lower rates of core knowledge. Participants who were unemployed had an OR of 0.616 demonstrating a higher rate of core knowledge when compared to those who were in white-collar occupations (OR = 0.616, 95% CI 0.417–0.910).

**Table 3:** Odds ratios and 95% confidence intervals for having a high cancer prevention and control core knowledge by sociodemographic and other factors in Fujian Province, China.

**Variable**	**Crude OR**	**Adjust OR**	**95%CI**
**Occupation (White collar as reference)**			
Blue collar	0.319	0.309	0.233–0.411
Unemployment	0.639	0.616	0.417–0.910
**Marital status (Living alone as reference)**			
Married	1.571	1.803	1.234–2.636
**Educational level (Bachelor's degree or higher as reference)**			
Primary school degree or below	0.258	0.199	0.121–0.326
Junior high school degree	0.440	0.306	0.195–0.480
Senior high school degree (including technical training)	0.760	0.623	0.399–0.972
**Family history of cancer (yes as reference)**			
Unclear	0.348	0.309	0.194–0.492
**Residence area (rural as reference)**			
Urban	1.897	1.789	1.405–2.278
**Self-evaluated health (very good as reference)**			
Average	1.922	1.954	1.459–2.617
Good	1.442	1.471	1.171–1.847
Age (years)	0.986	0.986	0.977–0.996

SE, standard error; OR, odds ratio; CI, confidence interval.

## 4. Discussion

Cancer prevention and control core knowledge is crucial as it influences individuals' attitudes and practices to improve health-seeking behaviors and thereby significantly reduces cancer burden (5). However, the current epidemiological research investigating cancer prevention knowledge is limited in China (5, 10–12). This population-based study aimed to better understand cancer prevention and control core knowledge levels and its influencing factors among urban and rural adults in Fujian, China. The findings show the overall average rate of core knowledge of cancer prevention and control measures among adults was 56.01%, lower than other similar studies (11, 12), but higher than studies with smaller sample sizes. Most importantly, the study's average knowledge rate is lower than the nationally expected knowledge rate, which is at 70% for the general Chinese population by 2022 as set out by the Chinese Department of Health 2017–2025 short-term and long-term plan for the prevention and treatment of chronic diseases (6). This finding provides evidence for the need to develop a campaign to enhance cancer prevention and control knowledge in Southeastern China.

Interestingly, we found that although most adults in our study knew the significance of cancer secondary prevention (73.75%), less than half had sufficient knowledge of many areas of cancer including early clinical symptoms of cancer, major risk factors for developing cancer (e.g., unhealthy lifestyle habits, infectious factors), early detection or preventive measures, or basic concepts about cancer. The findings indicate that in order to reduce the future cancer burden in China, increasing the level of public awareness of cancer primary and secondary prevention measures should remain a priority through the provision of detailed information on cancer risk factors, preventive measures, cancer symptoms or signs, in addition to identifying opportunities to change individual behaviors (14).

This study found a significant association between cancer prevention and control core knowledge levels and participants' area of residence, occupation, educational level, marital status, family history of cancer, and self-evaluated health status. Regarding occupation, the current evidence regarding the association between occupation and cancer core knowledge is mixed, which might be due to the various definitions and classifications of occupations (11, 15). In this study, adults with white-collar occupations were more likely to have higher rates of core knowledge than adults with blue-collar occupations, or those who were unemployed. The possible explanations include white-collar workers usually have higher educational levels (bachelor's degree or higher), focus more on their health status, have insurance coverage, and have more opportunities to access social resources and healthcare-related information, all of which help them improve their cancer core knowledge levels (11, 15). Furthermore, blue-collar participants are more likely to be exposed to hazards, such as dust and noise in construction work or ultraviolet radiation in welding work (5). Taken together, blue-collar workers with junior high school or less of education should be identified as a target population for community-level cancer prevention interventions.

In line with previous studies (5, 9, 11), we also found that residents in urban areas or with a family history of cancer were more likely to have a higher core knowledge rate. The differences seen when comparing areas of residence can be explained by the urban-rural disparity in access to health services and exposure to certain risk factors (20). Currently in China, urban-rural disparities are obvious, with people living in urban areas having greater cancer knowledge as well as higher cancer rates (21). In addition, a family history of cancer may reflect genetic as well as behavioral and environmental risks shared by family members (9). Health promotion theories, such as the Health Belief Model and protection motivation, predict that people are more likely to take preventive actions when they perceive their risk of negative health outcomes to be high (16). Furthermore, the Chinese philosophy of “destiny” may motivate people who have a family history of cancer to participate in emotional control, self-care activities, and active cancer prevention measures (22). Thus, the development of an effective cancer prevention program should consider cultural and geographical factors.

Results show a significantly higher core knowledge rate among married adults or people with a self-rated good or average health status. This is consistent with the Learning Partner Model (23), which claims that partners' adequate core knowledge transfers to others in their social network, mostly to their family, and thus improves others' core knowledge level of cancer prevention and control measures. A previous study found that married men's knowledge and support also has a positive effect on wives' cancer screening knowledge (24). As stated by the Salutogenic Model (25), people whose self-rated health levels were average or good can be motivated to increase their core knowledge in order to improve their self-care activities. Finally, the negative association between age and cancer knowledge might be explained by the older study participants being more likely to avoid any discussion of illness including cancer, owing to the fear of bringing about unlucky karma (22), therefore decreasing opportunities to gain cancer prevention knowledge.

There are some limitations of this study. Firstly, this study was a cross-sectional survey and therefore did not allow us to infer causality to explain the relationship between cancer prevention and control measures and core knowledge and socio-demographic characteristics. Thus, future longitudinal studies are needed to address this important issue. Secondly, study participants were recruited from one province which is not representative of the entire Chinese population. However, because this is a population sampling study, its results can inform basic strategies for stakeholders to use in designing a provincial intervention to enhance cancer prevention and control measures knowledge. Thirdly, our participants were not stratified by high- and low-risk groups, a stratification that should be considered by future related studies in order to provide a clearer picture of cancer prevention and control measures core knowledge.

## 5. Conclusion

The overall average rate of core knowledge of cancer prevention and control measures among adults in Fujian Province was below the 70% target set out by the Chinese Department of Health. Adults who were residents of urban areas, held white-collar jobs, married, had a bachelor's degree or above, a family history of cancer, and were self-rated as having a good health status were associated with a higher core knowledge level of cancer prevention and control measures. These findings may help healthcare providers and policy stakeholders design effective primary prevention interventions to enhance the general population's cancer prevention and control knowledge and subsequently decrease cancer burden.

## Data availability statement

The data analyzed in this study is subject to the following licenses/restrictions: the data that support the findings of this study are availabe from the corresponding author upon reasonable request. Requests to access these datasets should be directed to FH, pt860315@163.com.

## Ethics statement

The studies involving human participants were reviewed and approved by this study was approved by the Ethics Review Committee of Fujian Provincial Centers for Disease Control and Prevention (Grant Number: K2021-101-01). The patients/participants provided their written informed consent to participate in this study.

## Author contributions

FH and W-TC was responsible for the research design and revisions to the manuscript. TY contributed to data analysis and initial writing of the manuscript. XL and JL contributed to data collection and analysis. All authors have contributed to the conception and design of the study, drafted or have been involved in revising this manuscript, reviewed the final version of this manuscript before submission, and agree to be accountable for all aspects of the work.
