# Supplementary material for: Influencing factors of cancer prevention and control among urban and rural adults in Fujian, China: A cross-sectional survey
Source: Front Public Health. 2022 Dec 21;10:1053183. doi: 10.3389/fpubh.2022.1053183 (PMC9811185; doi:10.3389/fpubh.2022.1053183)
Supplement: Supplementary file 1 [file Table_1.DOCX]

Supplementary Material

**Appendix A. Cancer prevention and control core knowledge awareness rates (n=2074)**

| Survey # | Item | Awareness rate (%) |
| --- | --- | --- |
| C11 | What are Breast cancer warning symptoms? | 16.5% |
| C2 | Which of the following biological factors increases the risk of cancer？ | 22.3% |
| C9 | What's the correct description of cancer prevention and therapy? | 22.7% |
| C5 | Which of the following unhealthy lifestyle habits can increase the risk of cancer? | 26.7% |
| C14 | What's the correct description of cancer pain? | 27.2% |
| C13 | Who are high-risk populations for developing cancer? | 35.6% |
| C15 | What should cancer patients do to achieve better rehabilitation? | 45.6% |
| C12 | Which of the following symptoms may be a warning signal of cancer and should be investigated? | 47.9% |
| C1 | What is a cancer-related risk factor? | 48.8% |
| A6 | The type and stage of cancer have little effect on the treatment plan and can be treated directly according to the plan that works best for others. | 49.1% |
| C6 | What's a cancer prevention measure? | 50.0% |
| C10 | What's the correct description of the common cancer screening method? | 50.4% |
| A4 | At the recovery stage of cancer, maintaining a patient’s stable condition only needs actively adjust the body’s immunity, while a stable mindset has little effect. | 52.7% |
| A1 | Cancer usually takes ten decades to develop. | 53.2% |
| A12 | Cancer is not a terminal disease, and patients can "peacefully coexist" with it. | 53.5% |
| B10 | Is cancer contagious? | 54.2% |
| B8 | What's the correct description of cancer prevention? | 54.9% |
| B1 | What's the correct description of the relationship between cancer and lifestyle? | 55.0% |
| B5 | What's the correct description of cancer incidence in our country in recent years? | 55.0% |
| C8 | Which of the following measures can effectively reduce the incidence and mortality of cancer? | 57.0% |
| A10 | If there are warning signs such as "a mole on your body becomes darker and larger in a short period," you can remove the mole yourself or observe it at home for some time without going to the hospital immediately. | 58.0% |
| B9 | What's the correct description of cancer-related factors? | 59.1% |
| B11 | What's the correct description of an anti-cancer physical examination? | 63.3% |
| B19 | What's the best practice in response to positive results of an anti-cancer physical examination? | 65.0% |
| B17 | What's the correct description of the effectiveness of cancer treatments and survival time? | 66.2% |
| B12 | How frequently should one get a cancer check-up? | 66.4% |
| B20 | What's the correct measure for a patient needs to take cancer drugs for a long time? | 67.7% |
| B16 | What's the patient suffering from if frequent bowel movements occur recently and the stools are bloody and thin? | 67.9% |
| A9 | Regular dry cough, sputum with blood, and other symptoms suggest lung cancer may be present | 68.3% |
| A7 | Some cancer (e.g., breast cancer, bowel cancer) have certain transmissibility, so the family should cause to take them seriously. | 70.0% |
| B2 | What is the most common cancer in China? | 70.2% |
| B21 | What is the best practice for cancer patients after discharge from the hospital? | 70.3% |
| A5 | Vaccinations, such as the HPV vaccine, can prevent the development of certain cancers. | 71.6% |
| A11 | Many cancers can be cured by taking folk remedies, taking health supplements, or following advertised treatments | 72.8% |
| A2 | Cancer incidence and death rates have improved in some regions of the country, due to the adaption of cancer prevention, education, and screening measures | 74.9% |
| A13 | The most common first symptom of esophageal cancer is dysphagia. | 76.2% |
| A3 | In addition to surgical treatment, cancer treatment methods also include radiotherapy, chemotherapy, targeted therapy, immune therapy, endocrine therapy, and treatment of a combination of traditional Chinese and Western medicine. | 80.7% |
| A8 | Anti-cancer physical examination is an individual physical examination for common cancers and most cancers can be early detected by currently available medical measures. | 81.3% |
